# Developing a Rate Law for Ce(III) Oxidation by Manganese Oxides

**DOI:** 10.1021/acs.est.4c12688

**Published:** 2025-06-17

**Authors:** Hang Xu, Pan Liu, Simin Zhao, Yinghao Wen, Yuanzhi Tang

**Affiliations:** School of Earth and Atmospheric Sciences, 1372 Georgia Institute of Technology, 311 Ferst Drive, Atlanta, Georgia 30332-0340, United States

**Keywords:** Ce anomaly, rare earth elements, Mn oxides, Ce oxidation, kinetic rate law

## Abstract

Rare earth elements (REEs) are critical minerals that are indispensable for clean energy technologies. Understanding REE occurrence and transport in natural environments is important for the prediction and identification of REE resources. Cerium (Ce) is a rare earth element that exhibits multiple oxidation states. The oxidation of dissolved Ce­(III) by manganese oxides (MnO_2_) and the resulting Ce anomaly is used as an indicator for tracing biogeochemical processes controlling REE transport and mobility, as well as a paleo-redox proxy for understanding Earth’s oxygenation events. However, a detailed kinetic rate law for this process is still lacking. This study determines the reaction orders and rate constant for Ce­(III) oxidation by δ-MnO_2_ using the initial rate method. The overall reaction follows a first order for Ce­(III) and δ-MnO_2_ and a 0.5th order for OH^–^, resulting in an overall 2.5th order. The calculated overall rate constant (*k*) was 1.4 × 10^6^ L^3/2^ mol^–1/2^ g^–1^ h^–1^. Kinetic modeling was employed to distinguish Ce adsorption and oxidation by using redox-inert Ce-analogues La and Nd. Our experimental and kinetic modeling results suggest that Ce­(III) oxidation by δ-MnO_2_ occurs in multiple steps: the adsorption of Ce­(III) on the δ-MnO_2_ surface, the oxidation of Ce­(III), and surface precipitation of Ce^IV^O_2_. Our findings provide important insights into the quantitative applications of Ce anomaly as a proxy to investigate various biogeochemical processes.

## Graphical abstract

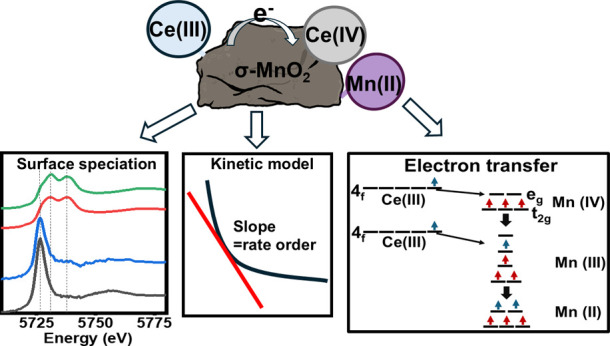

## 1 Introduction

Rare earth elements (REEs) are a group of 17 elements, including lanthanides, yttrium (Y), and scandium (Sc). They exhibit similar chemical behaviors across various geological and geochemical processes, such as weathering, particle scavenging, and sedimentation.^1^ REEs typically exist as dissolved trivalent cations in natural waters, except for cerium (Ce) and europium (Eu). Ce exists in two oxidation states (+3 and +4) due to its unique electronic configuration, where the single outer-shell electron in the Ce­(III) 4f orbital is more easily removed than in other REEs.^1^ This redox flexibility allows the oxidation of Ce­(III) to Ce­(IV) and subsequent precipitation as Ce^IV^O_2_ due to its low solubility.^2–5^ This redox-induced solubility difference governs the mobility and transport of Ce in natural settings. Ce­(III) oxidation can occur in the presence of naturally prevalent oxidants such as oxygen,^6^ Fe (oxyhydr)­oxides,^7–9^ and Mn oxides.^10^ Oxidation of Ce­(III) by these oxidants removes Ce from the dissolved pool and decouples it from the remaining trivalent REE. This process leads to a phenomenon called Ce anomaly, in which the Ce concentration is either depleted or enriched relative to other REEs.^11,12^

The unique redox sensitivity of Ce relative to other REE enables several applications, such as tracing various geochemical reactions, facilitating oxygen storage through the CeO_2_/MnO_2_ cathode catalyst, and serving as a paleo-redox proxy for interpreting the redox conditions in early Earth surface and marine environments.^13–15^ Reconstructing the oxygenation history of the Earth is critical for understanding early biogeochemical cycles and the search for life on exoplanets. There is an increasing number of studies developing and applying redox proxies.^4,16–19^ Ce anomaly presents several advantages over alternative proxies in investigating geochemical processes. First, the one-electron transfer between Ce­(III) and Ce­(IV), and the high redox potential (Ce^4+^ + e^–^ → Ce^3+^, *E* ^0^ = +1.72 V), makes Ce particularly sensitive to redox conditions compared to other proxies, such as MoO_4_^2–^/MoO_2_ (*E* ^0^ = +0.5 V) and Fe^3+^/Fe^2+^ (*E* ^0^ = +0.77 V).^20–23^ Second, high-resolution quantification of Ce anomaly and oxidation state characterization can be reliably achieved through inductively coupled plasma-mass spectrometry (ICP-MS) and synchrotron X-ray analysis.^10,24^ For example, Ce L_III_-edge X-ray absorption near edge structure (XANES) analysis can be employed to clearly distinguish Ce­(III) and Ce­(IV), with the Ce^IV^O_2_ spectrum displaying two distinct pre-edge peaks at 5370 and 5740 eV, compared to a single peak for Ce­(III) at 5715–5720 eV.^10^ Furthermore, Ce anomaly remains largely unaffected by physical and chemical processes such as early diagenesis,^25^ sediment transport and sorting,^26^ and mineral transformations.^26^ As a result, Ce anomaly can provide reliable geochemical information in marine sediments and mineral deposits, including redox state, trace element presence, and isotope ratios.^27,28^ For instance, a compilation of Ce anomaly data in well-preserved carbonate minerals was employed to track oceanic oxygenation history from the Neoproterozoic to the Phanerozoic.^29^ A follow-up study combining Ce anomaly with Ce modeling quantitatively constrained Paleoproterozoic atmospheric O_2_ as low as ∼0.1% of the present atmospheric level (PAL).^30^ A stable Ce isotope (δ ^142^Ce) has already been utilized as a paleo-redox proxy to trace the redox dynamics in various geological settings, such as ferromanganese deposits,^31^ upper continental crust,^12^ natural waters,^32^ and phosphorites.^33^ Ce anomaly serves as a sensitive indicator of redox conditions in shallow oceans that enables a comprehensive reconstruction of O_2_ variations from shallow ocean to deeper waters when employed along with other independent proxies.^6^

Additionally, Ce redox cycling is linked to the biogeochemical cycling of essential trace metals. In aquatic environments, accumulated Mn and Fe (oxyhydr)­oxides in sediments participate in Ce­(III) oxidation. These oxides provide large surface areas and abundant active sites, facilitating redox-driven, microbially mediated processes.^7,34^ A previous work had suggested that Ce coexists with Mn, Fe, and cobalt (Co) in oxygen-deficient zones (ODZs), where oxygen concentrations are extremely low in subsurface waters.^35^ Under such anoxic conditions, the dissolution of Mn and Fe oxides is mediated by Ce­(III) oxidation to release Mn­(II) and Fe­(II) from the water column. In ODZs, these redox-sensitive metals are mobilized from continental shelf sediments and transported via low-oxygen water masses into the oceans. These processes serve as important sources of trace metals to marine phytoplankton, potentially alleviating nutrient limitation, particularly for essential elements such as Fe.^36–38^ Upon reoxygenation of these sediments, the formation of Mn and Fe oxides leads to the oxidation of Ce­(III) to Ce­(IV), which is then transported into the sediments. Also, Ce­(III) can be preferentially scavenged by Mn-oxidizing bacteria to form Ce­(IV) in oxic environments, resulting in a depletion of Ce­(III) relative to its neighboring lanthanides (e.g., La, Pr, and Nd).^39^

Several previous studies have investigated the natural oxidants for Ce­(III) oxidation and the relevant reaction mechanism. Nakada et al. found that dissolved Ce­(III) was slowly oxidized to Ce­(IV) in pure O_2_ within 1 week.^40^ In contrast, the quasi-equilibrium of isotopic fractionation (^142^Ce/^140^Ce) driven by δ-MnO_2_-mediated Ce­(III) oxidation was reached in 6 h under the same conditions, suggesting the higher efficiency of δ-MnO_2_ in Ce­(III) oxidation than O_2_.^24^ Bau et al. reported that Ce­(IV) is associated with hydrous Mn oxides and Fe oxides in ferromanganese crusts, which indicates the potential catalytic effects of Mn/Fe oxides on Ce­(III) oxidation.^41^ Further observation showed that Ce anomalies were observed exclusively in the presence of Mn oxides and not in the presence of Fe oxides under well-controlled laboratory conditions.^24,42^ This may be attributed to the lower reduction potential of Fe­(III)/Fe­(II) (*E*° = +0.77 V) compared to Mn­(IV)/Mn­(III) (*E*° = +0.95 V).^43^ These findings suggest that Mn oxides demonstrate a higher efficiency in Ce­(III) oxidation compared to O_2_ and Fe (oxyhydr)­oxides, likely due to the high redox sensitivity and enhanced surface reactivity.^44–47^ This opens up questions regarding the reaction mechanisms and kinetics of Ce oxidation on Mn oxides (eq 1).^45^

$$2{\mathrm{Ce}}^{3+}+{\mathrm{MnO}}_{2}+4{\mathrm{OH}}^{-}\to 2{\mathrm{Ce}}^{4+}+{\mathrm{Mn}}^{2+}+2H_{2}O$$ 1

While some studies have investigated Ce oxidation as an important redox proxy and geological indicator, the rate law governing Ce oxidation by Mn oxides remains poorly defined. This is primarily due to the rapid nature of Ce oxidation by MnO_2_ ^24,45^ and fast electron transfer from Ce­(III) to MnO_2_ (*t*_1/2_ ≈ 50 min under comparable conditions), as well as the low solubility of Ce­(IV) (log *K*_sp_ CeO_2_ = −59.3 ± 0.31), which together challenge the distinction of reaction mechanisms and rates among Ce­(III) adsorption, Ce­(III) oxidation, and Ce­(IV) precipitation.^48,49^ Additionally, previous studies focused on a relatively narrow range of experimental conditions, such as constant pH or fixed reactant concentrations, such that the effects of reactants and pH conditions on the mechanism cannot be inferred.^44,45^ A systematic experimental approach incorporating all of the reactant variables and pH conditions is therefore needed. Furthermore, quantitative determination of reaction parameters using Ce anomaly data is still challenging due to limited understanding of the reaction mechanism and kinetic data.^30^

This study aims to address these knowledge gaps by elucidating the reaction mechanisms of Ce­(III) oxidation by Mn oxides and determining major kinetic parameters of this reaction. δ-MnO_2_ was selected as a representative Mn oxide phase because it is the most common fresh Mn oxide phase formed through various biotic and abiotic processes and is commonly found in freshwater, sediments, and soils.^50–53^ The effects and partial reaction orders of pH, initial Ce­(III) concentration, and δ-MnO_2_ loading on the kinetics of Ce­(III) oxidation were monitored. The adsorption and oxidation of Ce­(III) by δ-MnO_2_ were differentiated by employing redox-inert Ce-analogues La and Nd, which is useful in elucidating the geochemical behaviors of other redox-active metals in the natural environments. Ce­(III) oxidation was further confirmed by XANES analysis of the reaction products. The objectives of this study are to (i) establish a comprehensive rate law for Ce oxidation by synthesized δ-MnO_2_, and (ii) understand the effects of key geochemical constraints (pH, concentration of dissolved Ce, and δ-MnO_2_ loading) on the reaction rates. This work can contribute to a more comprehensive understanding of Ce oxidation by MnO_2_ and enable quantitative applications of Ce anomaly data in studying important biogeochemical processes.

## 2 Materials and Methods

### 2.1 Chemicals and Materials

All chemicals used were ACS grade or higher. Cerium­(III) chloride heptahydrate (CeCl_3_·7H_2_O), lanthanum­(III) chloride heptahydrate (LaCl_3_·7H_2_O), neodymium­(III) trichloride (NdCl_3_), indium internal standard (TraceCERT), REE mix standard (TraceCERT), 2-(4-(2-hydroxyethyl)­piperazin-1-yl)­ethanesulfonic acid (HEPES), sodium chloride (NaCl), sodium hydroxide (NaOH), and hydrochloric acid (HCl) were purchased from Sigma-Aldrich. High-purity γ-Al_2_O_3_ was purchased from Sky Spring Nanomaterials Inc. δ-MnO_2_ and ferrihydrite were synthesized following previous procedures (Text S1).^54^ All solids were repeatedly rinsed with DI water and freeze-dried. The structure of synthesized solids was confirmed by X-ray diffraction (XRD). Specific surface areas were determined by Brunauer–Emmett–Teller (BET) gas adsorption analysis using an Autosorb-1-MP surface pore analyzer (Quantachrome Corp.). The surface areas of δ-MnO_2_, γ-Al_2_O_3_, and ferrihydrite are 221.9 ± 1.3, 206, and 236 ± 1 m^2^/g, respectively.

### 2.2 Ce­(III) Adsorption and Oxidation by δ-MnO_2_

All experiments were conducted inside a Coy anaerobic glovebox (95% N_2_, 5% H_2_). To illustrate the redox conditions of the reaction system, a Eh–pH diagram was constructed for the CeO_2_/Ce^3+^ and MnO_2_/Mn^2+^ couples at 25 °C, calculated with the Nernst equation at 10 μM ion activities and plotted in Python 3.9 (Figure S1). Anoxic deionized (DI) water was prepared by sterilizing DI water under UV light for an hour, followed by boiling on a hot plate while under an ultrahigh-purity N_2_ gas purge. The water was then allowed to cool naturally under a N_2_ purge, then capped and transferred into the glovebox for experiments. Batch experiments on Ce­(III) oxidation by δ-MnO_2_ were conducted in the glovebox at room temperature. 0.1 M NaCl and 10 mM HEPES were first added into 30 mL flasks before the addition of synthetic δ-MnO_2_. The pH for each batch experiment was adjusted by using 0.1 mol/L NaOH and HCl. A CeCl_3_·7H_2_O stock solution (10 mM) was added afterward. The initial concentrations of Ce­(III) and δ-MnO_2_ were 200 μM and 0.1 g/L, respectively. The oxidation reactions were conducted for 120 min, based on preliminary tests, which allowed the reaction to reach equilibrium. A 3 mL aliquot of the reaction suspension was collected at 0, 5, 10, 20, 30, 45, 60, 90, and 120 min and filtered (0.2 μm) for analyses of dissolved Mn and Ce by ICP-MS.

### 2.3 Differentiating Ce­(III) Adsorption and Oxidation Using Ce-Analogues

To differentiate between the adsorption and oxidation of Ce on δ-MnO_2_, lanthanum (La) and neodymium (Nd) were used as the Ce-analogues. With their neighboring positions in the periodic table, La, Ce, and Nd have descending ionic radii (1.16, 1.14, and 1.11 Å, respectively).^55^ Additionally, they share nearly identical first-shell Ln^3+^–O distances (2.69, 2.67, and 2.61 Å) with the coordinated water molecule and comparable hydration energies (−54.97, −55.82, and −57.31 kcal mol^–1^).^56^ These similarities make them exhibit similar sorption behaviors on metal oxide (e.g., iron oxyhydroxide) and clay minerals (e.g., kaolinite and halloysite), forming surface complexes of comparable stability.^57,58^ Unlike Ce, La and Nd are not redox-active. We reasonably assumed that Ce, La, and Nd would exhibit comparable adsorption behaviors.^59^ Therefore, the average adsorption rate of Nd and La was used to represent that of Ce in this work. Batch adsorption experiments for La and Nd onto δ-MnO_2_ were conducted under the same conditions described in Section 2.2. Two Al and Fe oxide minerals, γ-alumina (γ-Al_2_O_3_) and ferrihydrite, were selected for comparison.^60^ γ-Al_2_O_3_ was obtained from Sigma–Aldrich and characterized in our previous work.^61^ Ferrihydrite was synthesized following an established procedure.^62^ For the sorption experiments, 200 μM Nd or La was mixed with 0.1 g/L γ-Al_2_O_3_, ferrihydrite, or δ-MnO_2_ suspension. Samples were collected using similar methods as described above and analyzed by ICP-MS.

### 2.4 Determining the Kinetics of Ce­(III) Oxidation by δ-MnO_2_

Batch experiments were designed to determine the partial orders of the reaction for each reactant in Ce­(III) oxidation by δ-MnO_2_. The kinetic rate law was investigated under the following conditions: (i) varied initial concentrations of Ce­(III) (50–300 μM) at pH 6.5 with 0.1 g/L δ-MnO_2_, (ii) varied initial δ-MnO_2_ loading (0.05–0.3 g/L) at pH 6.5 and a fixed Ce­(III) concentration of 200 μM, and (iii) varied pH (5.5–7.5) with 200 μM Ce­(III) and 0.1 g/L δ-MnO_2_. Reactions were carried out for 10 min, and samples were collected at 0, 0.5, 1, 1.5, 2, 2.5, 3, 4, 5, and 10 min to analyze dissolved Mn and Ce concentrations using ICP-MS. Initial reaction rate (*R*_0_) was calculated based on the change in concentration of Ce­(III) via linear regression. Experimental data were interpreted using the initial rate method, which is useful for exploring interfacial reaction mechanisms such as kinetics of adsorption,^63^ ligand binding,^64^ and isotopic exchange.^65^ This method assumes that the rate of reaction at the early stages is unaffected by significant changes in the reactant concentrations, thus avoiding complications arising from product inhibition or from any subsequent reactions.^66^

### 2.5 Kinetic Modeling Using MATLAB

To predict the change in speciation and elucidate the reaction mechanism, kinetic modeling was performed under the following conditions: 10 mM NaCl, 200 μM Ce­(III), pH 6.5, and 0.1 g/L δ-MnO_2_. All data was coded in MATLAB version R2022b, and the changes in the concentration of different Ce and Mn species over time were predicted. The two analogues La and Nd were selected as proxies for Ce based on previous studies demonstrating their similar REE–O distances in the first coordination shell and identical coordination numbers, which suggests comparative adsorption behaviors. This rationale supports their use in distinguishing Ce­(III) adsorption from oxidation when no alternative approach is available.^67,68^ The average adsorption rates of La and Nd on δ-MnO_2_ were thereby selected to represent that of Ce. Corrcoef function was employed to assess the goodness of fit between experimental and modeling results. Parameters and equations used in kinetic modeling are described in Text S2.

### 2.6 Analytic Methods

The solution concentrations of REE were determined by ICP-MS (Text S3). Ce speciation in the reaction products was characterized by X-ray absorption spectroscopy (XAS) at Advanced Photon Source (APS) Beamline 5-BM-D (Text S4). XAS data analysis used the software Athena.^69^ XRD analysis was conducted to identify the formation of new phases (Text S5).

## 3 Results and Discussion

### 3.1 Ce­(III) Adsorption and Oxidation by γ-Al_2_O_3_, Ferrihydrite, and δ-MnO_2_

To examine Ce­(III) adsorption and oxidation under different conditions, three common metal oxide phases, γ-Al_2_O_3_, ferrihydrite, and δ-MnO_2_ with similar surface areas ranging from 200 to 240 m^2^ g^–1^, were used in batch sorption experiments under anoxic conditions. Similar batch experiments were performed using La­(III) and Nd­(III) as Ce­(III) analogues to probe the Ce­(III) adsorption reaction. It is noted that under the pH range and redox condition of this study, Ce­(III) and Ce­(IV) are the dominant Ce species in the aqueous and the solid phase, respectively.^70^ The mass balance was calculated using the following equation: Total Ce = Adsorbed Ce­(III) + Oxidized Ce­(IV) + Remaining aqueous Ce­(III). The adsorption kinetics of Ce­(III) were approximated by averaging the experimental data of La­(III) and Nd­(III).

Our results showed that the adsorption of Ce­(III), La­(III), and Nd­(III) on γ-Al_2_O_3_ was negligible, and their adsorption on ferrihydrite is in the range of ∼20–50% (Figure S2a). The adsorption of Ce­(III) was similar to and between those of La­(III) and Nd­(III) as expected, which demonstrates the feasibility of using La­(III) and Nd­(III) as Ce­(III) proxies. For γ-Al_2_O_3_ and ferrihydrite, the aqueous concentration of Ce­(III) did not significantly differ from those of La­(III) and Nd­(III) (Figure S2a,b). This suggests that Ce­(III) oxidation on these mineral surfaces was negligible, as Ce­(III) oxidation and the formation of insoluble Ce­(IV) would have resulted in a decreased Ce­(III) concentration compared to those of La­(III) and Nd­(III). In contrast, dissolved Ce­(III) concentration decreased markedly in the presence of δ-MnO_2_, resulting in near-complete removal within 20 min (Figure S2c). Based on the measured data of La­(III) and Nd­(III), the adsorption of Ce­(III) onto δ-MnO_2_ was estimated to account for only ∼10% of the Ce­(III) removal, whereas the remaining 90% was attributed to Ce­(III) oxidation by δ-MnO_2_. Among these three oxide minerals, our results suggest that γ-Al_2_O_3_ and ferrihydrite possess negligible effects on Ce­(III) oxidation (Figure S2a,b), whereas δ-MnO_2_ is highly effective (Figure S2c). To further confirm this, the reacted solids were analyzed by Ce L_III_-edge XANES. The XANES spectra of Ce­(III) and Ce­(IV) displayed distinct features. The different electronic configurations of Ce­(III) and Ce­(IV) (4f^1^ and 4f^0^, respectively) result in the greater effective nuclear charge in Ce­(IV), which shifts the Ce­(IV) absorption edge to a higher energy than Ce­(III) in the XANES spectrum, typically by about 5–10 eV.^71^ Moreover, the Ce­(III) spectrum has a single peak associated with the final state 2p^5^-4f^1^-5d*, while Ce­(IV) presents double peaks corresponding to the final states 2p^5^-4f^2^-5d* and 2p^5^-4f^0^-5d*.^72,73^ The solid products from the Ce­(III) + δ-MnO_2_ treatment group displayed spectral features similar to those of the reference compound Ce^IV^O_2_ (Figure 1), indicating Ce­(IV) formation in the presence of δ-MnO_2_. The formation of CeO_2_ was further confirmed by XRD (Text S5 and Figure S3). In contrast, the Ce L_III_-edge XANES spectra of solid products from the other treatments showed the main edge at 5726.5 eV, suggesting negligible Ce­(III) oxidation, which was consistent with the aqueous solution data.

**Figure 1:**
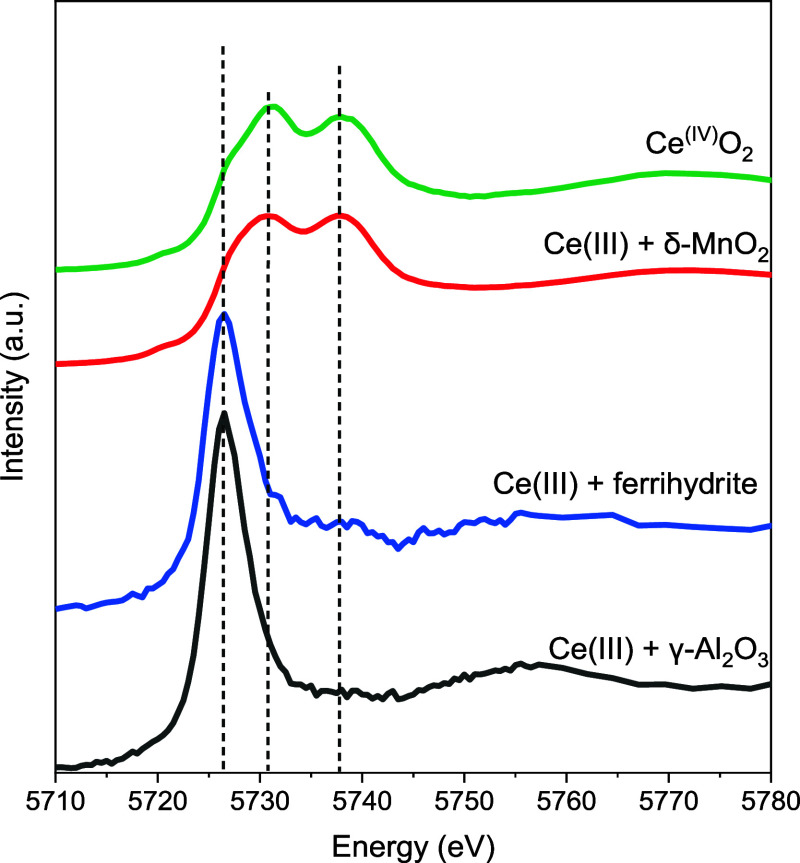
Ce L_III_-edge XANES spectra of the final products of Ce­(III) interaction with γ-Al_2_O_3_, ferrihydrite, or δ-MnO_2_, as well as the reference compound Ce^(IV)^O_2_.

### 3.2 Speciation of Ce and Mn

To unravel the reaction mechanisms of Ce­(III) oxidation by δ-MnO_2_, experiments were conducted by reacting 200 μM Ce­(III) with 0.1 g/L δ-MnO_2_ at pH 6.5. The results showed a rapid decrease in dissolved Ce­(III) concentration, with complete removal by 120 min (Figure 2). Concurrently, an increase in dissolved Mn­(II) was detected, suggesting the reductive dissolution of δ-MnO_2_ and the release of Mn­(II) during Ce­(III) oxidation. Interestingly, the observed ratio of changes in aqueous Ce­(III) and Mn­(II) concentrations $\left(\frac{d[\mathrm{Ce}(\mathrm{III})]}{d[\mathrm{Mn}(\mathrm{II})]}\right)$ was slightly lower than the reaction stoichiometry in eq 1. This was possibly due to the adsorption of released Mn­(II) onto MnO_2_,^53^ coprecipitation of Mn­(II) with Ce­(IV),^74–78^ and/or adsorption of Mn­(II) to Ce^IV^O_2_.^79^ Since the change in Mn­(II) concentration did not accurately represent the amount of reduced MnO_2_, change in dissolved Ce­(III) concentration was monitored in subsequent experiments to examine the kinetics of Ce­(III) oxidation.

**Figure 2:**
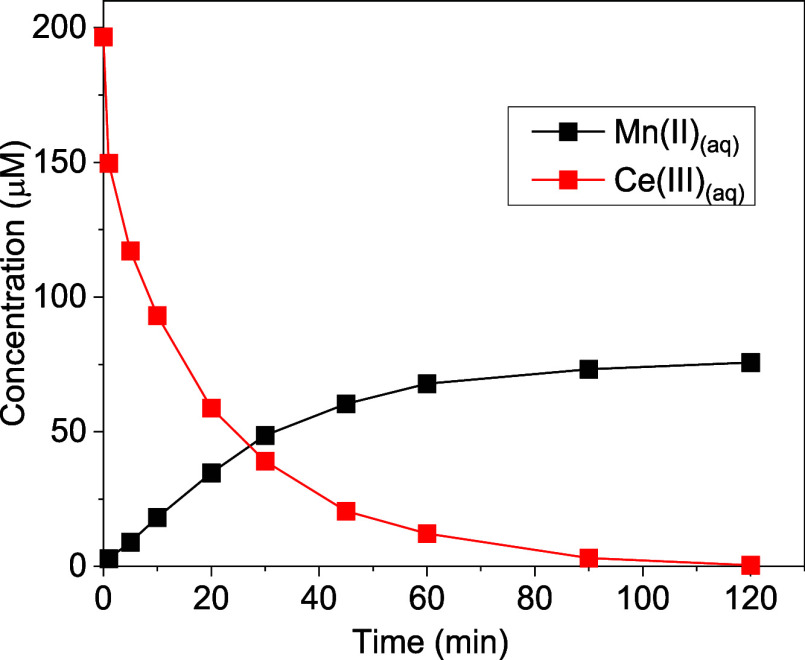
Concentrations of dissolved Ce­(III) and Mn­(II) over time. (Experimental condition: 10 mM NaCl, 200 μM initial Ce­(III), pH 6.5 (10 mM HEPES), δ-MnO_2_ loading 0.1 g/L).

### 3.3 Reaction Orders for Ce­(III) Oxidation by δ-MnO_2_

The general rate law for the oxidation of Ce­(III) by MnO_2_ (eq 1) is expressed as a function of Ce­(III), δ-MnO_2_, and OH^–^ concentrations (eq 2) as follows:

$$R=-\frac{d[\mathrm{Ce}(\mathrm{III})]}{dt}=k[\mathrm{Ce}(\mathrm{III}){]}^{a}[\mathrm{Mn}O_{2}{]}^{b}[{\mathrm{OH}}^{-}{]}^{c}$$ 2

where *R* is the rate of the reaction, *k* is the reaction rate constant, and [Ce­(III)], [MnO_2_], and [OH^–^] are the concentrations of Ce­(III), δ-MnO_2_, and OH^–^, respectively. The partial orders of reaction for each reactant are represented as *a*, *b*, and *c*, respectively. Based on the results from Section 3.2, a reaction time of 2 min was the most linear range for initial rate order kinetics of Ce­(III) (Figure 2), which was used in the subsequent experiments for initial *R* determination.

The evolution of Ce­(III) during the kinetic experiments at varied initial Ce­(III) concentrations, δ-MnO_2_ loading, and pH are shown in Figure 3a–c, with all experiments and conditions detailed in Table 1. Consistent with the above discussions, the oxidation of Ce­(III) by δ-MnO_2_ was rapid within the initial 10 min across all experiments. The initial Ce­(III) concentration, OH^–^ concentration, and δ-MnO_2_ loading all exhibited positive correlations with the reaction rates. Moreover, the concentration of Ce­(III) displayed a consistent exponential decay pattern throughout all experiments, indicating a pseudo-first-order reaction. All of the derivations of the rate law were based on this condition. The replicate of batch experiments is displayed in Figures S4–S7, suggesting consistent results and supporting the reliability of the experiments.

**Figure 3:**
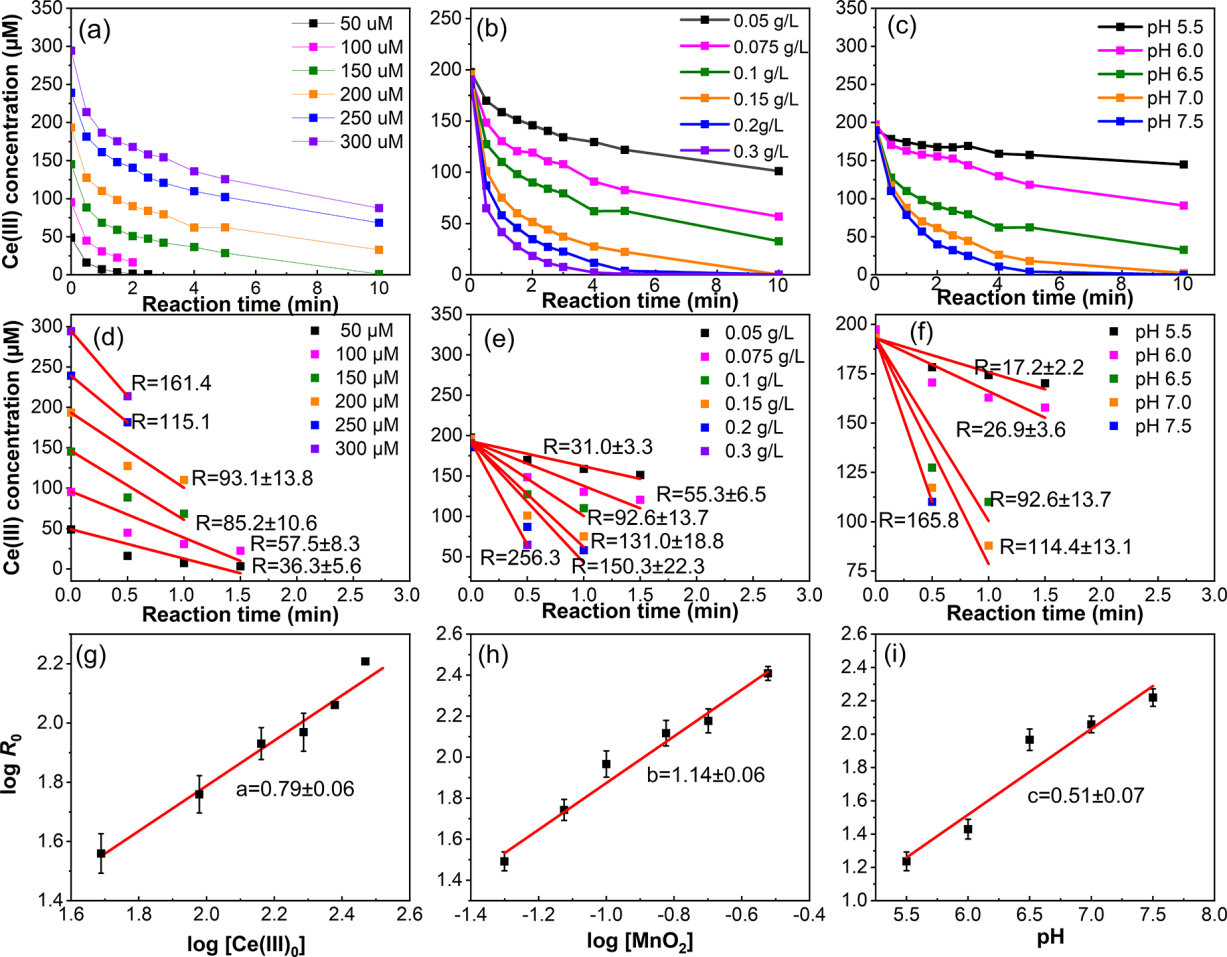
Evolution of Ce­(III) concentration over time for the experimental group with varied initial Ce­(III) concentrations (50–300 μM) (a), δ-MnO_2_ loadings (0.05–0.3 g/L) (b), and pH values (5.5–7.5) (c). (d–f) Evolution of Ce­(III) concentration during the first 2 min of reaction to determine the initial reaction rate (*R*_0_). (g–i) Log initial rate of Ce­(III) oxidation (*R*_0_) vs log of initial Ce­(III), δ-MnO_2_ concentration, and the negative of the pH value to determine the partial order of the reaction.

**Table 1:** Experimental Conditions and Kinetic Modeling Results, Including pH, Initial Ce­(III) Concentration, δ-MnO_2_ Loading, Observed Rate Constant (*k*_obs_), Initial Rate (*R*_0_), and Overall Rate Constant (*k*).

experimental group	pH	[Ce(III)]_0_ (μM)	[MnO_2_]_0_ (g L^–1^)	*k*_obs_ (h^–1^)	*R*_0_ (μM h^–1^)	*k* (L^3/2^ mol^–1/2^ g^–1^ h^–1^)
(I) varied initial Ce(III) concentration	6.5	50	0.01	43.56	2178 ± 336	2449558.81 ± 94473.34
		100		32.28	3450 ± 498	1940077.57 ± 140022.99
		150		27.62	5112 ± 636	1916459.24 ± 178824.54
		200		27.93	5586 ± 828	1570619.32 ± 232809.31
		250		34.08	6906	1553411.68
		300		34.50	9684	1815237.80
(II) varied MnO_2_ loading	6.5	200	0.05	9.30	1860 ± 198	1045954.87 ± 55671.79
			0.75	16.59	3318 ± 390	124389.90 ± 109656.56
			0.1	27.78	5556 ± 822	1562184.20 ± 231122.28
			0.15	39.30	7860 ± 1128	1473334.27 ± 317160.51
			0.2	45.09	9018 ± 1338	1267798.512 ± 376206.35
			0.3	76.89	15378	1441280.82
(III) varied pH	5.5	200	0.01	5.16	1032 ± 110	917592.18 ± 97805.36755
	6			8.07	1614 ± 180	807000.00 ± 90000.00
	6.5			27.78	5556 ± 685	1562184.20 ± 192601.90
	7			34.32	6864 ± 655	1085293.69 ± 103564.59
	7.5			49.74	9948	884516.18 ± 97805.37

#### 3.3.1 Partial Order of Reaction for [Ce­(III)]

To investigate the order of reaction for [Ce­(III)], varied initial Ce­(III) concentrations ([Ce­(III)]_0_) were examined while fixing δ-MnO_2_ loading (0.1 g/L) at pH 6.5 in 10 mM HEPES. The initial reaction rates (*R*_0_) with different initial Ce­(III) concentrations were calculated via linear regression (Figure 3d), and the rate law can be simplified and expressed using the order constant *k*_obs1_ in eq 3, where *k*_obs1_ = *k*[MnO_2_]*^b^* [OH^–^]*^c^*.

$$R_{0}=-\frac{d[\mathrm{Ce}(\mathrm{III})]}{dt}=k_{\mathrm{obs}1}[\mathrm{Ce}(\mathrm{III}){]}^{a}$$ 3

Taking the logarithm of eq 3 results in eq 4:

$$\log(R_{0})=\log(k_{\mathrm{obs}1})+a\;\log([\mathrm{Ce}(\mathrm{III})])$$ 4

which is shown in Figure 3g to determine the partial order for Ce­(III). A slope of 0.79 ± 0.06, rounded up to 1, indicates a partial order of 1 for [Ce­(III)].

#### 3.3.2 Partial Order of Reaction for [MnO_2_]

To determine the partial order of reaction for [MnO_2_], experiments were conducted by varying the initial δ-MnO_2_ loading ([MnO_2_]_0_) while fixing the initial 200 μM Ce­(III) concentration to pH 6.5. Similar to Section 3.3.1, *R*_0_ with different δ-MnO_2_ loadings was calculated using eq 5 (Figure 3e).

$$R_{0}=-\frac{d[\mathrm{Ce}(\mathrm{III})]}{dt}=k_{\mathrm{obs}2}[{\mathrm{MnO}}_{2}{]}^{b}$$ 5

where *k*_obs2_ = *k*[Ce­(III)]­[OH^–^]*^c^*. Equation 6 was then linearized in its logarithmic form to determine both the pseudo-first-order rate constant (*k*_obs2_) and the reaction order via linear regression (eq 6):

$$\log(R_{0})=\log(k_{\mathrm{obs}2})+b\;\log([{\mathrm{MnO}}_{2}])$$ 6

The obtained slope was 1.14 ± 0.06 (Figure 3h), indicating a partial order of 1 for [MnO_2_].

#### 3.3.3 Partial Order of Reaction for [OH^–^]

Batch experiments were also conducted at varied pH (5.5–7.5), with 200 μM Ce­(III) and 0.1 g/L δ-MnO_2_ to determine the partial order of reaction for [OH^–^]. The corresponding order rate law is shown as

$$R_{0}=-\frac{d[\mathrm{Ce}(\mathrm{III})]}{dt}=k_{\mathrm{obs}3}[{\mathrm{OH}}^{-}{]}^{c}$$ 7

where the pseudo-first-order rate constant *k*_obs3_ = *k*[Ce­(III)]­[MnO_2_], and *R*_0_ with different [OH^–^] is shown in Figure 3f. Equation 7 can be converted into eq 8 to calculate *k*_obs3_.

$$\log(R_{0})=\log(k_{\mathrm{obs}})+c\;\log([{\mathrm{OH}}^{-}])=\log(k_{\mathrm{obs}})-14c+c\cdot \mathrm{pH}$$ 8

Based on linear regression, coefficient *c* was calculated to be 0.51 ± 0.07 (Figure 3i), indicating a partial order of 0.5 for [OH^–^]. This finding suggested that the effect of [OH^–^] (or pH) on the kinetics of this reaction is less pronounced compared to [Ce­(III)] and [MnO_2_], which agrees with a previous study.^80^ The pH primarily influences the reaction rate of Ce­(III) oxidation by altering the surface charge of δ-MnO_2_ and speciation of Ce. δ-MnO_2_ typically has two p*K*_a_ values: −1.6 and 4.6.^81–83^ Within the pH range in this work (5.5–7.5), δ-MnO_2_ undergoes deprotonation and results in a negative surface charge that promotes the adsorption of metal cations.^83^ In addition, Ce predominantly exists as dissolved Ce­(III) from pH 2–8 and gradually transforms to Ce­(OH)^2+^, Ce­(OH)_3(s)_, and Ce­(OH)_4_^–^ as the pH continues to increase based on thermodynamic calculations.^40^ Throughout the pH conditions in this study, electrostatic attraction can facilitate the adsorption of positively charged Ce­(III) onto negatively charged δ-MnO_2_ and subsequent oxidation.^84^

### 3.4 Overall Rate Law and Rate Constant

To verify the derived rate law, similar experiments and derivations were repeated, which yielded consistent results (Table S1 and Figures S4–S7). Putting all of the results together, the oxidation of Ce­(III) by δ-MnO_2_ is proposed to follow an overall rate order of 2.5. Specifically, as shown in eq 9, it follows first-order kinetics with respect to [Ce­(III)] and [MnO_2_] and a 0.5th order with respect to [OH^–^]. The rate law (eq 9) and initial reaction rate (*R*_0_) were used to determine the corresponding reaction rate constants (*k*_obs1_ *–k*_obs3_) (Table 1). Using this approach, an overall reaction rate constant of 1377464.31 ± 523295.46 L^3/2^·mol^–1/2^·g^–1^·h^–1^ was obtained by averaging the *k* from all experiments performed in this study.

$$R=-\frac{d[\mathrm{Ce}(\mathrm{III})]}{dt}=k[\mathrm{Ce}(\mathrm{III})][{\mathrm{MnO}}_{2}][{\mathrm{OH}}^{-}{]}^{0.5}$$ 9

Table 2 summarizes the reaction conditions and estimated Ce­(IV) fractions from previous studies. Since no *k*_obs_ values were reported in previous studies, we instead compared the experimental conditions and reaction periods with those of this study. Previous research indicated that O_2_ and Fe (oxyhydr)­oxide had minimal impacts on Ce­(III) oxidation, while the presence of MnO_2_ resulted in a noticeable Ce anomaly and an increased Ce­(IV) fraction, which agrees with our findings. However, differences in the reaction rate were observed, where a complete removal of dissolved Ce­(III) was achieved within 90 min in the presence of δ-MnO_2_ in this study (Figure S2c), contrasting with the previously reported Ce­(IV) fraction of ∼0.32–1 observed over extended periods of 6–168 h.^24,40,43,45,85^

**Table 2:** Summary of Previous Studies on Ce Oxidation by Three Natural Oxidants: O_2_, Fe (Hydro)­oxides, and Mn Oxides^a^

oxidant	oxidant concentration	Ce(III) (μM)	background electrolyte or buffer	pH	time	estimated Ce(IV) fraction	ref
O_2_	O_2_ gas (bubbling)	N/A	N/A	5.0	1 w	0.03–0.12	Nakada et al.^24^
	O_2_ gas (bubbling)	71.4, 143	0.7 M NaCl, 2.25 mM NaHCO_3_	6.8–11.0	1 w	0.27–0.97	Nakada et al.^40^
	ambient air	1000	20 mM MES	6.0	24 h	0	Zheng et al.^44^
	ambient air	1000	20 mM MES, 20–200 mM CeO_2_	6.0	24 h	∼0.5–0.9	Zheng et al.^44^
Mn oxide, ambient air	∼0.01 g/L δ-MnO_2_	0.89	0.1–0.7 M NaNO_3_, NaCl, or Na_2_SO_4_	4–9	72 h	0.5–1*	De Carlo and Wen^43^
	0.0032 g/L δ-MnO_2_	14.3	0.5 M NaCl	4.7–6.8	130 h	∼>0.95*	Ohta and Kawabe^45^
	0.0040 g/L δ-MnO_2_	∼7	0.1 or 0.7 M NaCl	5.0	6 h	0.32–1.0	Nakada et al.^24^
	0.17 g/L MnO_2_	10, 100	0.01 M NaCl, 0.033–0.33 g/L microbial cells	3–8	168 h	0.5–0.8	Ohnuki et al.^85^
	δ-MnO_2_, loading N/A	7.14–143	0.7 M NaCl, 2.25 mM NaHCO_3_	6.8–11.0	6 h	0.21–0.99	Nakada et al.^40^
	1 mM biogenic Mn oxides	1000	20 mM MES	6.0	24 h	0.98	Zheng et al.^44^
Fe oxide, ambient air	∼0.01 g/L FeOOH	0.89	0.1–0.7 M NaNO_3_, NaCl, or Na_2_SO_4_	4–9	72 h	0*	De Carlo and Wen^43^
	∼0.006 g/L Fe oxyhydroxide	0.0001	0.1 M HCl titrated with NH_4_OH; artificial natural water	3.6–6.2	5 h	∼0.03	Bau^9^
	Fe oxyhydroxide, loading N/A	14.3	0.5 M NaCl	4.7–6.8	130 h	∼0.1*	Ohta and Kawabe^45^
	0.0097 g/L	∼61	0.1 or 0.7 M NaCl	5.0	6 h	0	Nakada et al.^24^
	ferrihydrite, loading N/A	7.14–143	0.7 M NaCl, 2.25 mM NaHCO_3_	6.8–11.0	6 h	∼>0.2 as Ce(III) > 71.4 uM at pH 8.2*	Nakada et al.^40^

a N/A, information not available.

Such a difference might be due to the following factors. First, Ce oxidation is influenced by the structure and characteristic of Mn oxides such as active surface sites and surface area.^83^ For example, compared to freshly synthesized δ-MnO_2_, aged δ-MnO_2_ (>2 years) only induced subtle or no positive Ce anomaly.^43^ Future studies are warranted to investigate the kinetics of Ce­(III) oxidation by biogenic and naturally occurring Mn oxides. Second, the batch experiments in this study were performed under well-controlled laboratory conditions without the interference of other complex environmental processes.^32,86,87^ Complexation of Ce by various organic and inorganic ligands was reported to either hinder or enhance Ce­(III) oxidation, yet a quantitative understanding of ligand effects on Ce oxidation kinetics is still lacking. Specifically, inorganic (e.g., Cl^–^, CO_3_^2–^, SO_4_^2–^) and organic ligands with varying affinities for Ce can influence the mobility and transport of Ce in natural settings.^43,88,89^ For example, NO_3_^–^considerably inhibited the oxidative adsorption of Ce­(III) on MnO_2_, while SO_4_^2–^ slightly enhanced it.^43^ However, the underlying mechanisms for such a distinct effect were unclear. De Carlo et al. (1997) postulated that SO_4_^2–^ could promote the electron transfer from Ce­(III) to Mn­(IV) by forming a ternary complex of Mn­(IV)-SO_4_-Ce­(III).^43^ Tanaka et al. demonstrated that the Ce­(IV) released from biogenic Mn oxides might preferentially form soluble complexes with organic molecules (40 and <670 kDa fractions) released by Mn­(II)-oxidizing microorganisms under circumneutral pH conditions (6.5–7.0).^88^ On the other hand, the complexation of Ce­(III) with natural organic matters such as humates remarkably suppressed the oxidation of Ce­(III) by Mn oxides.^90^ This is likely attributed to (i) the shielding of humate molecules on MnO_2_ surfaces and the scavenging of electrons,^90,91^ and/or (ii) the reduced availability of Ce­(III) due to the complexation by humates.^90^ In order to fully understand the environmental behaviors of Ce and other related processes, combined experimental and theoretical modeling investigations are desired to unveil the impacts of various inorganic and organic ligands on Ce­(III) sorption and oxidation.

### 3.5 Proposed Reaction Mechanisms and Kinetic Modeling

Based on the results, the process of Ce­(III) oxidation by δ-MnO_2_ is illustrated in Figure 5. First, Ce­(III) is adsorbed onto the surface of δ-MnO_2_, possibly via electrostatic interactions or surface complexation. Once adsorbed, Ce­(III) is oxidized by δ-MnO_2_, which leads to the subsequent precipitation of CeO_2_ (Figure S2) and release of Mn­(II) into the solution. Our results cannot rule out the possible surface complexation of Ce­(IV)–MnO_2_ under low Ce concentrations, although it was not observed in our reaction conditions. Romanchuk et al. found Ce­(IV)–MnO_2_ complexation formed at pH 2.5 with ppb-level Ce concentration using computational simulation.^10^ A previous study on U­(IV) adsorption had developed surface complexation models that account for both U­(IV) adsorption and precipitation on minerals, suggesting possible similar behavior with Ce­(IV).^92^ A recent study employed an element-specific surface crystallography method and successfully identified the surface complex of REE on alumina surfaces.^93^ Thus, future research might consider advanced methods, such as in situ XAS and thermodynamic modeling, to better characterize surface complexation, more comprehensively. A portion of the produced Mn­(II) then adsorbs onto the remaining Mn oxides and/or newly formed CeO_2_, while the remaining Mn­(II) is released to the aqueous phase. In addition, since Ce showed a distinct partition coefficient (solid-to-solution ratio) due to rapid Ce­(III) oxidation by δ-MnO_2_ and precipitation of CeO_2_,^91,94^ it can be difficult to distinguish Ce­(III) adsorption and oxidation. The average adsorption rate of two Ce­(III) analogues, La­(III) and Nd­(III), was thus used in the subsequent kinetic modeling to better differentiate between adsorption and oxidation processes.

Kinetics modeling was conducted using MATLAB and compared to experimental data (Figures 4 and S8). The modeling was based on the mass balance equations in Text S2. The modeling predicted a rapid oxidation of Ce­(III), leading to the formation of Ce­(IV). Initially, the fraction of adsorbed Ce­(III) increased, consistent with our proposed mechanism that dissolved Ce­(III) is first adsorbed onto the δ-MnO_2_ surface. However, the amount of adsorbed Ce­(III) began to decrease after ∼2 min, which resulted from several possibilities. First, the oxidation of Ce­(III) outpaced its adsorption, rapidly forming CeO_2_ (Figures 4a and S3). Second, the nucleation of Ce-bearing phases and the gradual dissolution of Mn oxide reduced (Figure 4b) the availability of surface sites, further hindering Ce­(III) adsorption. The corrcoef fitting coefficients are 0.9854 for the remaining Ce­(III) and 0.9897 for dissolved Mn­(II), indicating that the experimental and modeling results are in good agreement with each other.

**Figure 4:**
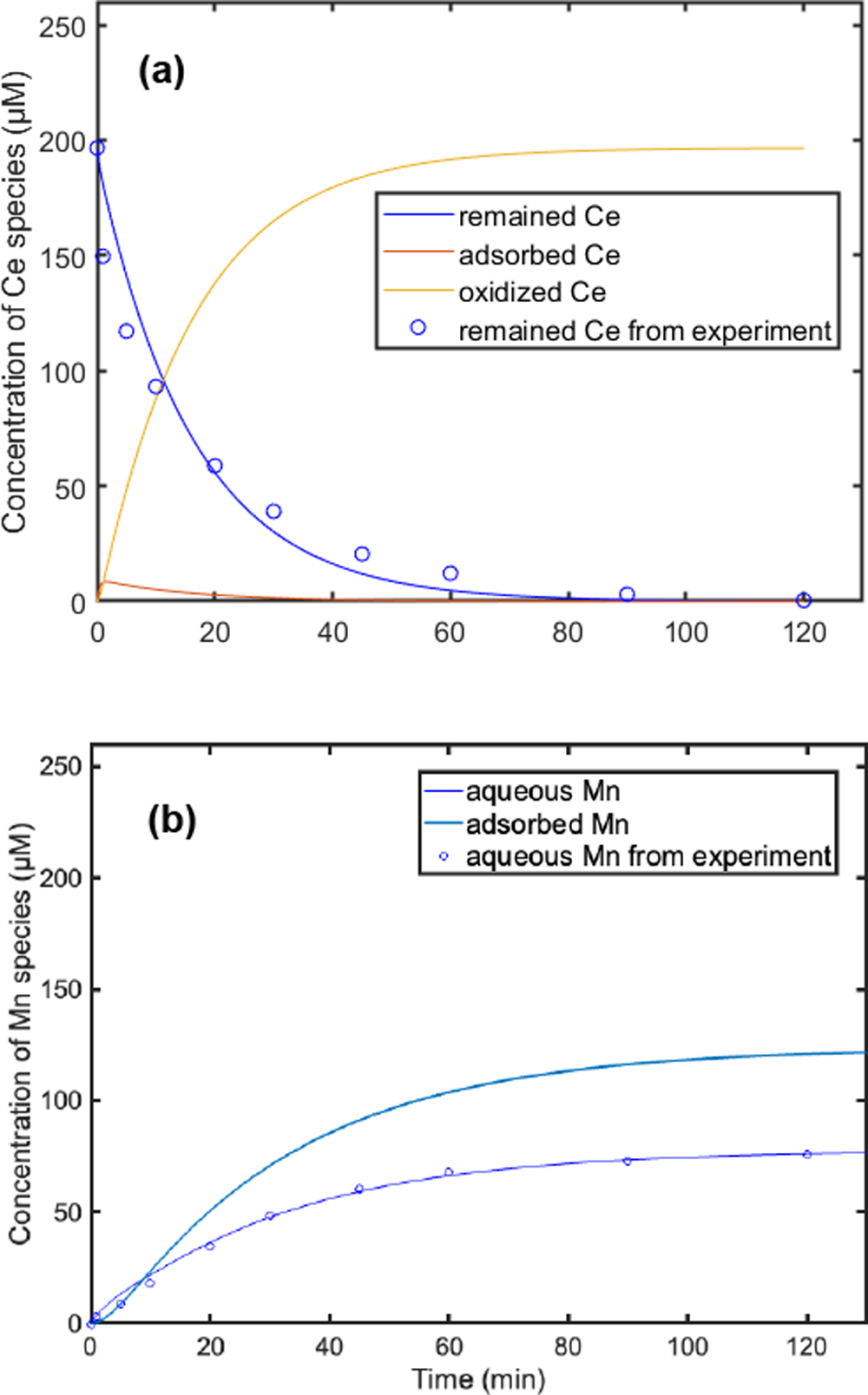
Kinetic modeling (lines) of experimental data (open circles) under experimental conditions of 10 mM NaCl, 200 μM initial Ce­(III), pH 6.5, and δ-MnO_2_ loading of 0.1 g/L. A duration of 5–30 min is selected for studying the oxidation rate. (a) Changes in Ce speciation; (b) changes in Mn speciation.

Putting these results together, we discuss the reaction mechanisms in two aspects. First, the standard redox potentials (*E* ^0^) for the Ce­(IV)/Ce­(III) and Mn­(IV)/Mn­(II) couples are ∼1.7 and ∼1.2 V, respectively.^95^ Such an *E* ^0^ difference leads to Δ*G* ^0^ = −*nF*Δ*E* ^0^ ≈ −9.1 × 10^4^ J mol^–1^, indicating the reaction is thermodynamically favored. Second, considering molecular symmetry, Ce­(III) 4f orbitals are more localized compared to Ce­(IV). This localization suggests that Ce­(III) 4f orbitals might ineffectively overlap with Mn­(IV) orbitals and make direct electron transfer unlikely.^96^ Instead, the reaction likely proceeds via surface complexation followed by electron transfer.^83,97^ We propose that Ce­(III) forms a precursor complex on the MnO_2_ surface before transferring electrons to Mn­(IV). In this complex, surface oxygen atoms can act as effective bridges. Their 2p orbitals are more spatially extended and possess symmetry compatibility with both the localized Ce­(III) 4f orbital and the empty Mn­(IV) orbital.^98^ This arrangement allows oxygen to accept an electron from Ce­(III), thereby stabilizing the extra electron density and forming the Ce^3+^–O–Mn^4+^ precursor complex. Subsequently, the electron density localized on oxygen is transferred to the Mn empty e_g_ orbital. Two possible electron transfer pathways are proposed. In the first, two Ce­(III), each using an oxygen atom as a bridge, donate electrons to a single Mn (IV) center (Figure 5a). This pathway is reported to be promoted by the presence of an orbital band on the MnO_2_ surfaces.^78^ Second, the stepwise two electron transfer pathways might occur, where Mn­(IV) accepts one electron into e_g_ orbitals and reduces it to Mn­(III). Mn­(III), with a d^4^(t^3^_2g_e^1^_g*_) electron configuration, is labile and undergoes Jahn–Teller distortion, leading to rapid ligand exchange. This allows a second one-electron transfer to form Mn­(II) (Figure 5b). Both pathways likely occur and fit the observed stoichiometries of Ce­(III) and Mn­(II) in Figure 1. These mechanistic interpretations are based on thermodynamic and molecular orbital considerations, but direct experimental evidence for the electron transfer process remains absent in prior studies.

**Figure 5:**
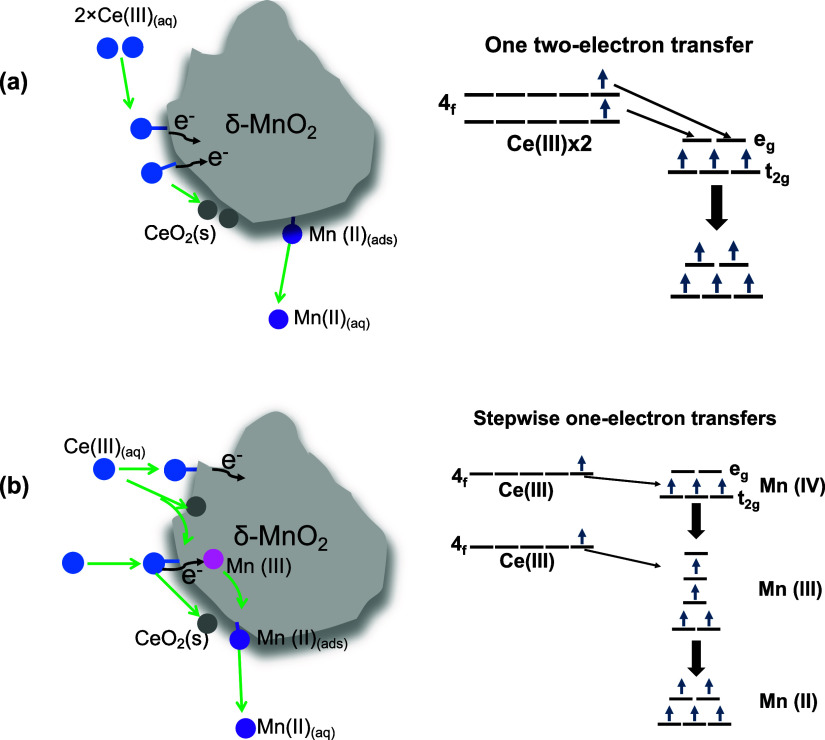
Schematic illustration showing the processes of Ce oxidation by δ-MnO_2_. (a) One two-electron transfer: electron transfer from Mn­(IV) atoms accepts one electron each from the lone electronic pair on Ce­(III); and (b) stepwise one-electron transfers: electron transfer from one Ce­(III) molecule to one Mn­(IV) atom, followed by Mn­(III) accepting one electron from the other Ce­(III), resulting in one Mn­(II) molecule.

### 3.6 Environmental Implications

Mn oxide minerals are ubiquitous in various natural environments, such as aquatic systems, soils, and sediments. Due to their redox activity, they play central roles in a variety of biogeochemical processes, such as controlling heavy metal speciation and mobility, serving as electron acceptors for microbial anaerobic respiration, coupling with carbon, sulfur, and nitrogen cycles, etc.^62,99–102^ Owing to their prevalence and high reactivity, Mn oxides can significantly impact the geochemical behaviors of Ce compared to other common oxidants including Fe (hydro)­oxides and dissolved oxygen. In this study, we investigated the kinetics of Ce­(III) oxidation by δ-MnO_2_ under the controlled experimental conditions. By employing nonredox-active REE analogues, we differentiated the adsorption and oxidation of Ce­(III) by δ-MnO_2_. This distinction allowed us to determine key kinetic parameters and derive a rate law for Ce­(III) oxidation.

Ce anomaly is commonly reported across various natural settings, such as ferromanganese nodules and crust, terrestrial soils and sediments, regolith, etc.^103,104^ In modern marine systems, Ce­(III) can be oxidized at oxic seawater–particle interfaces and immobilized as Ce­(IV) in ferromanganese nodules, producing strong Ce anomalies.^103^ Our kinetic data provide a kinetic basis for quantifying Ce removal fluxes, improving redox-cycling models for Fe and Mn, and informing Ce-based proxies for reconstructing ocean oxygenation events. Additionally, in terrestrial environments such as lateritic weathering profiles, alternating redox conditions were found to promote Ce­(III) oxidation and fixation as Ce­(IV) in Fe-rich horizons.^104^ Integrating the rate law developed in this study into reactive-transport models enables estimating the time scales of Ce accumulation and tracking redox evolution during soil formation. Moreover, in REE-enriched regolith deposits, Ce enrichment often coincides with the depletion of other REE,^105^ and the kinetic insights can support evaluating, interpreting, and predicting REE distribution in natural REE reservoirs.

Furthermore, the methodology developed for rate law derivation provides a framework for kinetic modeling. However, we acknowledge that there are other factors not considered, for example, variations in the Mn oxide crystal structure, ligand effects, and fluctuations in redox conditions. Future studies can adopt this framework to incorporate these additional environmental factors for characterizing and predicting Ce behaviors in different environmental settings such as sedimentary deposits and aqueous geochemical systems. Moreover, while no pseudo-first-order rate constant has been reported previously and batch experiments have generally been conducted under fixed conditions, our work considers the major constraints on the reaction, including pH and reactant concentrations, to determine both the reaction order and the rate constant. The faster oxidation rate predicted by our model suggests that in natural systems, where environmental conditions fluctuate and equilibrium may not always be attained, even transient changes in environmental conditions can induce significant shifts in the Ce­(III) oxidation state and affect its mobility. Consequently, the observed Ce anomalies may reflect recent environmental disturbance, underscoring the need for careful interpretation of the Ce anomaly record. Complexities in natural systems (e.g., redox cycling, ligand complexation, biological factors, etc.) warrant future research aiming at refining the kinetic framework.

## Supplementary Material



## Notes

Synthesis of δ-MnO_2_ and ferrihydrite (Text S1); kinetic modeling (Text S2); ICP-MS (Text S3); XAS (Text S4); XRD (Text S5); experimental conditions for replicating experiments (Table S1); Eh–pH diagram for the Ce–Mn redox system at 25 °C across pH 5.5–9 (Figure S1); La, Nd, and Ce uptake in the presence of γ-Al_2_O_3_, ferrihydrite, and δ-MnO_2_ (Figure S2); XRD pattern and phase identification of reacted samples and pure δ-MnO_2_ (Figure S3); evolution of Ce­(III) in the second replicate experiment (Figure S4); initial reaction rate (*R*_0_) in the second replicate experiment (Figure S5); third replicate to examine the reaction order with respect to pH (Figure S6); fourth replicate experiment to examine the reaction order with respect to pH (Figure S7); and modeling of Ce­(III) adsorption to δ-MnO_2_ (Figure S8) (PDF)
