# Supplementary material for: Developing a Rate Law for Ce(III) Oxidation by Manganese Oxides
Source: Environ Sci Technol. 2025 Jun 17;59(25):12606–17. doi: 10.1021/acs.est.4c12688 (PMC12224318; doi:10.1021/acs.est.4c12688)
Supplement: Supplementary file 1 [file es4c12688_si_001.pdf]

Supporting Information

Developing a Rate Law for Ce(III) Oxidation by Manganese Oxides

Hang Xu[#], Pan Liu[#], Simin Zhao[#], Yinghao Wen, Yuanzhi Tang*

School of Earth and Atmospheric Sciences

Georgia Institute of Technology

311 Ferst Dr, Atlanta, GA 30332-0340, USA

* Corresponding author.

Email: yuanzhi.tang@eas.gatech.edu

[#]Authors contributed equally.

Total pages: 14
Total text: 5
Total tables: 1
Total Figures: 8

Table of Contents

Text S1. Synthesis of δ-MnO_2 and ferrihydrite
Text S2. Kinetic modeling
Text S3. Inductively coupled plasma mass spectrometry (ICP-MS)
Text S4. X-ray absorption spectroscopy (XAS)
Text S5. X-ray diffraction (XRD)
Table S1. Experimental conditions for replicating experiments
Figure S1. Eh–pH diagram for the Ce–Mn redox system at 25 °C across pH 5.5–9.
Figure S2. La, Nd, and Ce uptake in the presence of γ-Al_2O_3, ferrihydrite, and δ-MnO_2
Figure S3. X-Ray diffraction pattern and phase identification of reacted samples and pure δ-MnO_2
Figure S4. Evolution of Ce(III) in second replicate experiment
Figure S5. The initial reaction rate (R_0) in second replicate experiment
Figure S6. Third replicate to examine the reaction order with respect to pH
Figure S7. Fourth replicate experiment to examine the reaction order with respect to pH
Figure S8. Modeling of Ce(III) adsorption to δ-MnO_2

Text S1. Synthesis of δ-MnO_2 and ferrihydrite

δ-MnO_2 was synthesized following the method described in our previous work.[1] A total of 32 mL of 0.3 M $MnSO_4$ solution was pumped into a reactor at a flow rate of 1 mL/min, where it reacted with 32 mL of 0.2 M $KMnO_4$ and 36 mL of 0.5 M NaOH at 5 Hz magnetic stirring. The resulting suspension was centrifuged and rinsed with deionized (DI) water, followed by dialysis until the conductivity of solution reached 2 µS/cm. The average oxidation state (AOS) of δ-MnO_2 was determined to be 3.8 using linear combination fitting (LCF) of Mn K-edge XANES spectra. Ferrihydrite was prepared according to our previous work.[2] Briefly, 2 L of DI water was heated to 75 °C in an oven, then 20 g $Fe(NO_3)_3 \cdot 9H_2O$ was dissolved with rapid stirring. The solution was returned to the oven until the color changed from gold to dark reddish brown, then rapidly cooled in ice water. The wet paste was transferred to a dialysis bag for three days, with the water changed several times each day. All synthesized materials were freeze-dried and characterized using X-ray diffraction (XRD) and Brunauer–Emmett–Teller (BET) surface area analysis.

Text S2. Modeling and Results

Rate laws of Ce and Mn species:

$$\frac{d[\mathrm{Ce(III)}]_{\mathrm{aqs}}}{dt} = -k_{\mathrm{Ce(III)ads}}[\mathrm{Ce(III)}]_{\mathrm{ads}} + k_{\mathrm{des}}[\mathrm{Ce(III)}]_{\mathrm{ads}}$$

$$\frac{d[\mathrm{Ce(III)}]_{\mathrm{ads}}}{dt} = k_{\mathrm{Ce(III)ads}}[\mathrm{Ce(III)}]_{\mathrm{ads}} - k_{\mathrm{Ce(III)obs}}[\mathrm{Ce(III)}]_{\mathrm{ads}} - k_{\mathrm{Ce(III)des}}[\mathrm{Ce(III)}]_{\mathrm{ads}}$$

$$\frac{d[\mathrm{Mn(II)}]_{\mathrm{aqs}}}{dt} = -k_{\mathrm{Mn(II)ads}}[\mathrm{Mn(II)}]_{\mathrm{aqs}} + k_{\mathrm{Mn(II)des}}[\mathrm{Mn(II)}]_{\mathrm{ads}}$$

$$\frac{d[\mathrm{Mn(II)}]_{\mathrm{ads}}}{dt} = \frac{1}{2}k_{\mathrm{Ce(III)obs}}[\mathrm{Ce(III)}]_{\mathrm{ads}} + k_{\mathrm{Mn(II)ads}}[\mathrm{Mn(II)}]_{\mathrm{aqs}} - k_{\mathrm{Mn(II)des}}[\mathrm{Mn(II)}]_{\mathrm{ads}}$$

Mass balance:

$[\mathrm{Ce(IV)}] = [\mathrm{Ce(III)}]_0 - [\mathrm{Ce(III)}]_{\mathrm{ads}} - [\mathrm{Ce(III)}]_{\mathrm{aqs}}$

$[\mathrm{Mn(II)}]_{\mathrm{aqs}} = [\mathrm{Mn(II)}]_{\mathrm{aqs0}} + [\mathrm{Mn(II)}]_{\mathrm{ads0}} - [\mathrm{Mn(II)}]_{\mathrm{ads}}$

Parameters:

$k_{\mathrm{Ce(III)ads}}$: rate constant for Ce adsorption
$k_{\mathrm{Ce(III)des}}$: rate constant for Ce desorption
$k_{\mathrm{Ce(III)obs}}$: rate constant for Ce oxidation
$k_{\mathrm{Mn(II)ads}}$: rate constant for Mn adsorption
$k_{\mathrm{Mn(II)des}}$: rate constant for Mn desorption
$[\mathrm{Ce(III)}]_{\mathrm{aqs}}$: dissolved Ce
$[\mathrm{Ce(III)}]_{\mathrm{ads}}$: adsorbed Ce(III)
$[\mathrm{Ce(III)}]_0$: dissolved Ce at beginning
$[\mathrm{Ce(IV)}]$: oxidized Ce
$[\mathrm{Mn(II)}]_{\mathrm{aqs}}$: dissolved Mn
$[\mathrm{Mn(II)}]_{\mathrm{ads}}$: adsorbed Mn
$[\mathrm{Mn(II)}]_{\mathrm{aqs0}}$: aqueous Mn at beginning
$[\mathrm{Mn(II)}]_{\mathrm{ads0}}$: adsorbed Mn at beginning

Text S3. Inductively coupled plasma mass spectrometry (ICP-MS)
Metal concentrations in collected samples were measured using ICP-MS (Agilent 7500a). All samples were prepared using 2% HNO_3 (v/v) as the matrix and spiked with 10 ppb of indium (In) as an internal standard. A series of calibration standards (0–200 ppb REE) was prepared using purchased REE standard (SPEX CertiPrep Inc.), and each standard solution was spiked with 10 ppb of In as well. The ICP-MS was carefully tuned for high sensitivity, low isobaric interference (CeO^+/Ce^+ <1%), and low doubly charged ions (<2%) before measurement. ^{139}La, ^{140}Ce, ^{143}Nd, ^{145}Nd, and ^{146}Nd, were measured simultaneously to ensure accuracy.

Text S4. X-ray Absorption Spectroscopy (XAS)
Ce L_{III}-edge X-ray absorption near edge structure (XANES) analysis was conducted on the solid products after sorption experiments. Samples were collected and dried in an anaerobic glove box. The dried solids were loaded onto epoxy sealed Kapton capillary tubes and maintained in anoxic conditions inside heat-sealed porches before X-ray absorption spectroscopy (XAS) measurements. XAS data was collected at Beamline 5-BM-D at the Advanced Photon Source (APS) at Argonne National Laboratory, Lemont, IL, USA. Multiple scans (4–6) were collected for each sample, averaged, and normalized.

Text S5. X-Ray Diffraction (XRD)
Mineralogical characterization of the reacted solids was performed using a Rigaku MiniFlex Powder X-ray Diffractometer equipped with a Cu Kα radiation source (λ = 1.54 Å) and a PIXcel 3D detector. Pure δ-MnO_2 Samples collected from reactions with initial Ce concentrations of 50 and 200 µM at pH 5.5 and 7.0 were analyzed to determine mineralogical transformations. XRD patterns were recorded over a 2θ range of 5−90° with a step size of 0.01° and a dwell time of 15 s per step under operating conditions of 45 kV and 40 mA. The collected data was processed in Jade software to identify crystalline phases, providing insights into potential Ce-bearing solid phases and secondary mineral formation under the experimental conditions.

Table S1. Reaction rate constants for replicate experimental conditions to examine the reaction order with respect to pH, at a fixed initial concentration of 200 μM Ce(III) and 0.1 g/L δ-MnO_2.

Condition	pH	$[Ce(III)]_0$ (μM)	$[MnO_2]_0$ (g L^{-1})	k_{obs} (h^{-1})	R_0 (μM h^{-1})	k ($L^{3/2}$ $mol^{-1/2}$ g^{-1} h^{-1})
**Second time pH varied**	5.5	200	0.1	2.60	520.80	463063.96
	6			6.41	1281.00	640500.00
	6.5			18.59	3717.00	1045111.35
	7			28.02	5604.00	886070.20
**Third time pH varied**	6.5	200	0.1	4.10	820.80	230582.44
	7			13.75	2749.32	434705.66
	7.5			26.52	5304.78	471669.05
	8			37.17	7433.40	371670.00

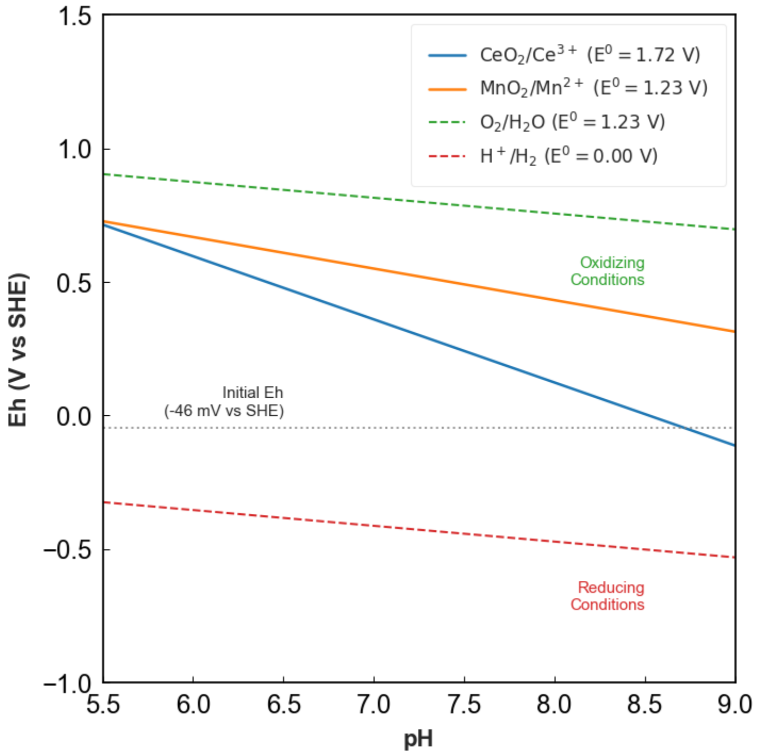

Figure S1. Eh–pH diagram for the Ce–Mn redox system at 25 °C, calculated for 10 µM activities of Ce^{3+} and Mn^{2+} across pH 5.5–9.

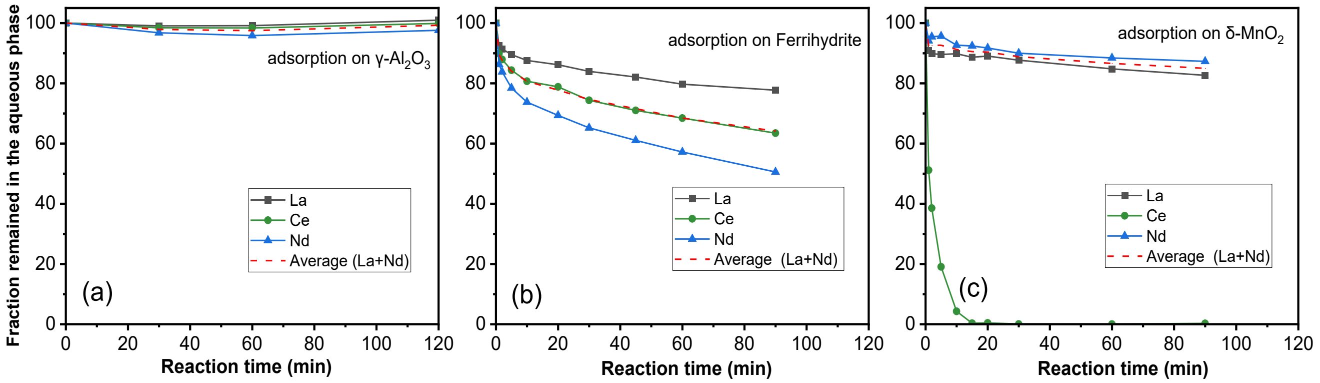

Figure S2. Ce(III) uptake under anoxic condition in the presence of (a) γ-Al_2O_3, (b) ferrihydrite, and (c) δ-MnO_2. In the γ-Al_2O_3 system, minimal La, Ce, and Nd adsorption and Ce oxidation were observed. In the ferrihydrite system, the observed decrease in La and Nd concentrations were due to adsorption as they are not redox sensitive under the experimental conditions. The calculated amount of Ce adsorption = (La adsorption + Nd adsorption)/2 is consistent with the observed amount of Ce uptake. Note: the red dash line represents the average of remained La(III) and Nd(III) percentage after adsorption.

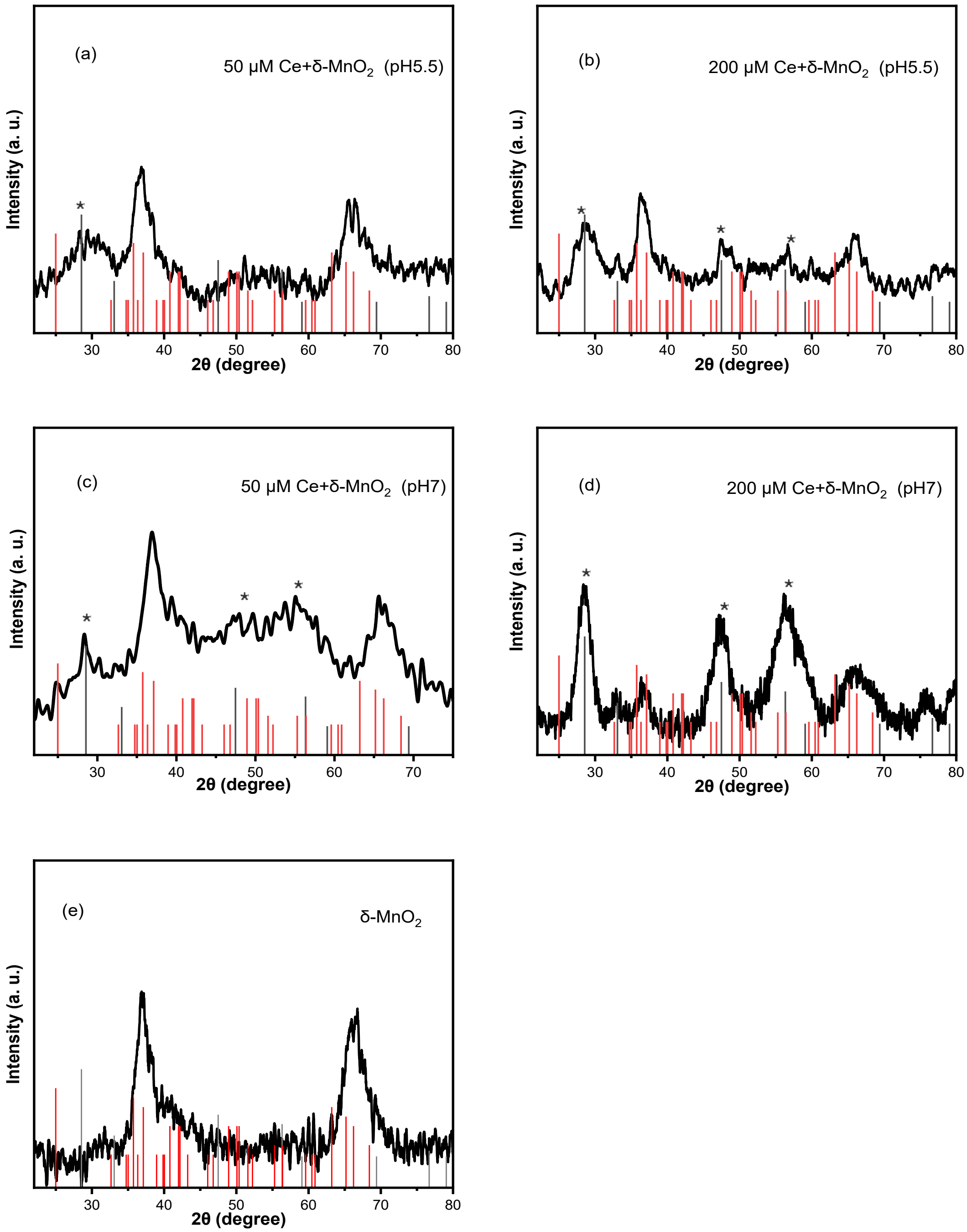

Figure S3. X-Ray diffraction pattern of (a)-(b) 0.1 g/L δ-MnO_2, 50 μM or 200 μM Ce initial concentrations at pH 5.5, respectively; (c)-(d) 0.1 g/L δ-MnO_2, 50 μM or 200 μM Ce initial concentrations at pH 7.0, respectively; (e) unreacted δ-MnO_2. (Red vertical lines is PFD#23-1046 of δ-MnO_2, black vertical lines is PFD#34-0394 of CeO_2, asterisks indicates CeO_2 peaks identified in the reacted samples).

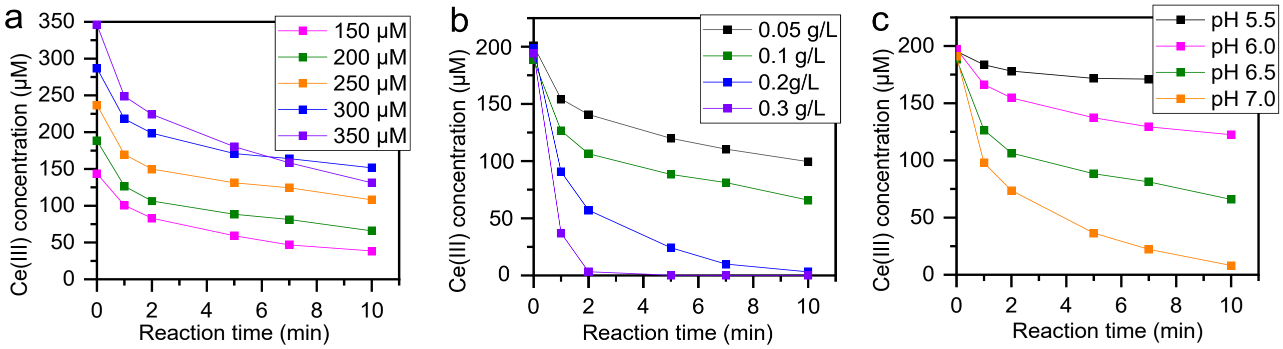

Figure S4. Evolution of Ce(III) in a replicate experiment to examine the reaction order with respect to Ce(III) concentration, δ-MnO_2 loading, and pH.

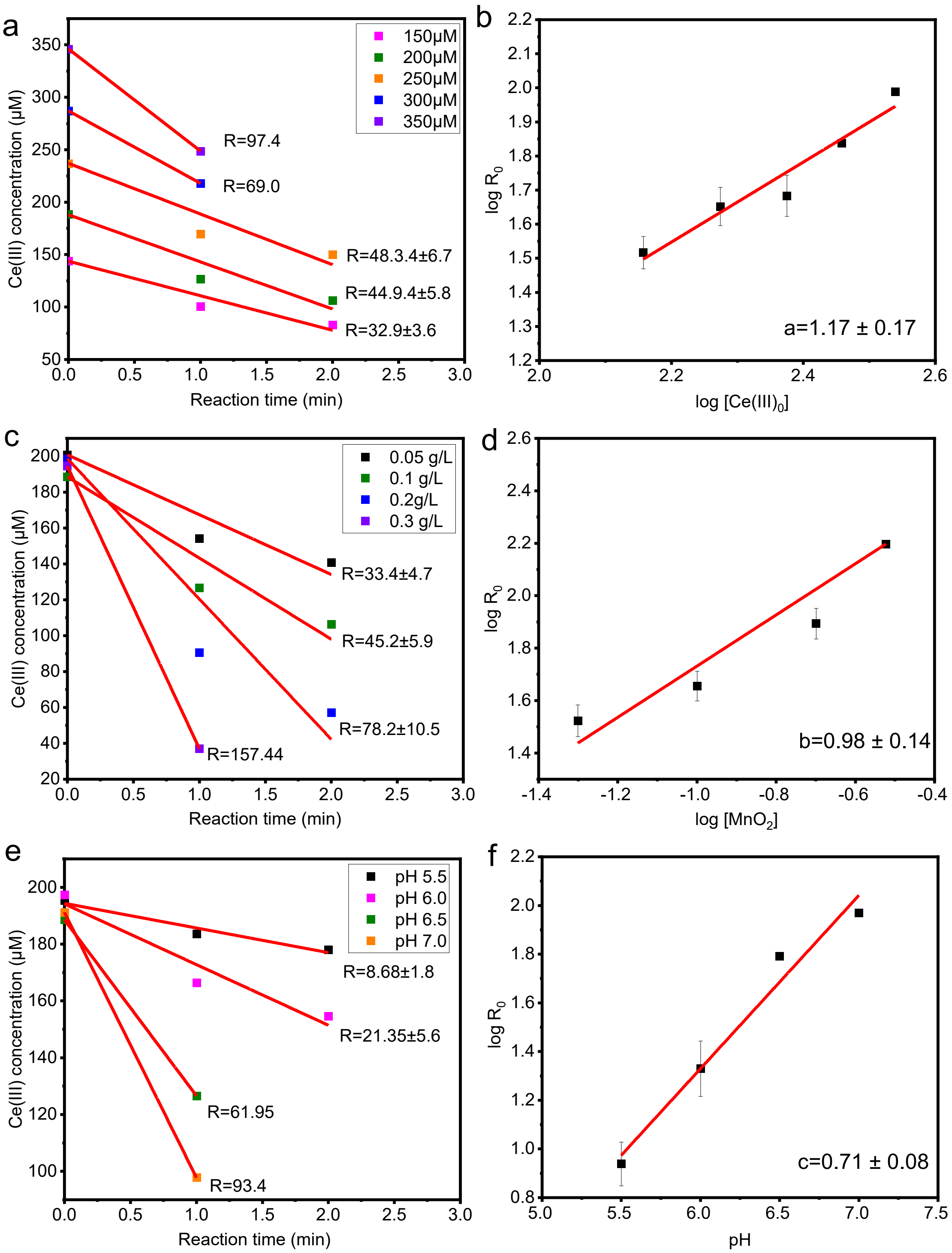

Figure S5. Results of replicate experiment to examine the reaction order with respect to Ce(III) concentration, δ-MnO_2 loading, and pH. Evolution of Ce(III) concentration during the first 2 min of reaction to determine the initial reaction rate (R_0).

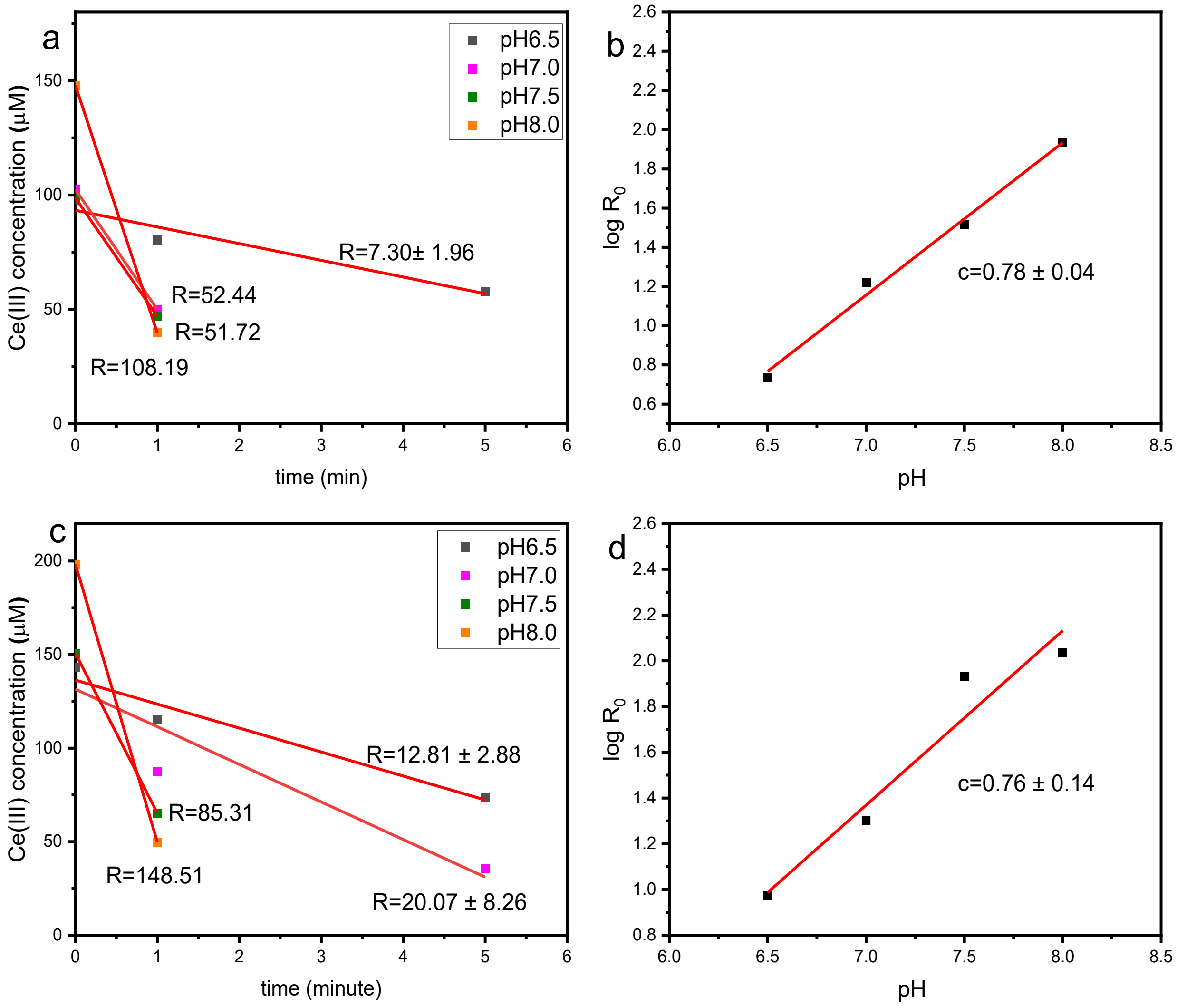

Figure S6. Results of replicate experiment to examine the reaction order with respect to pH at fixed δ-MnO_2 loading (0.1 g/L) and initial Ce(III) concentration (100 μM for (a) and (b) and 150 μM for (c) and (d)).

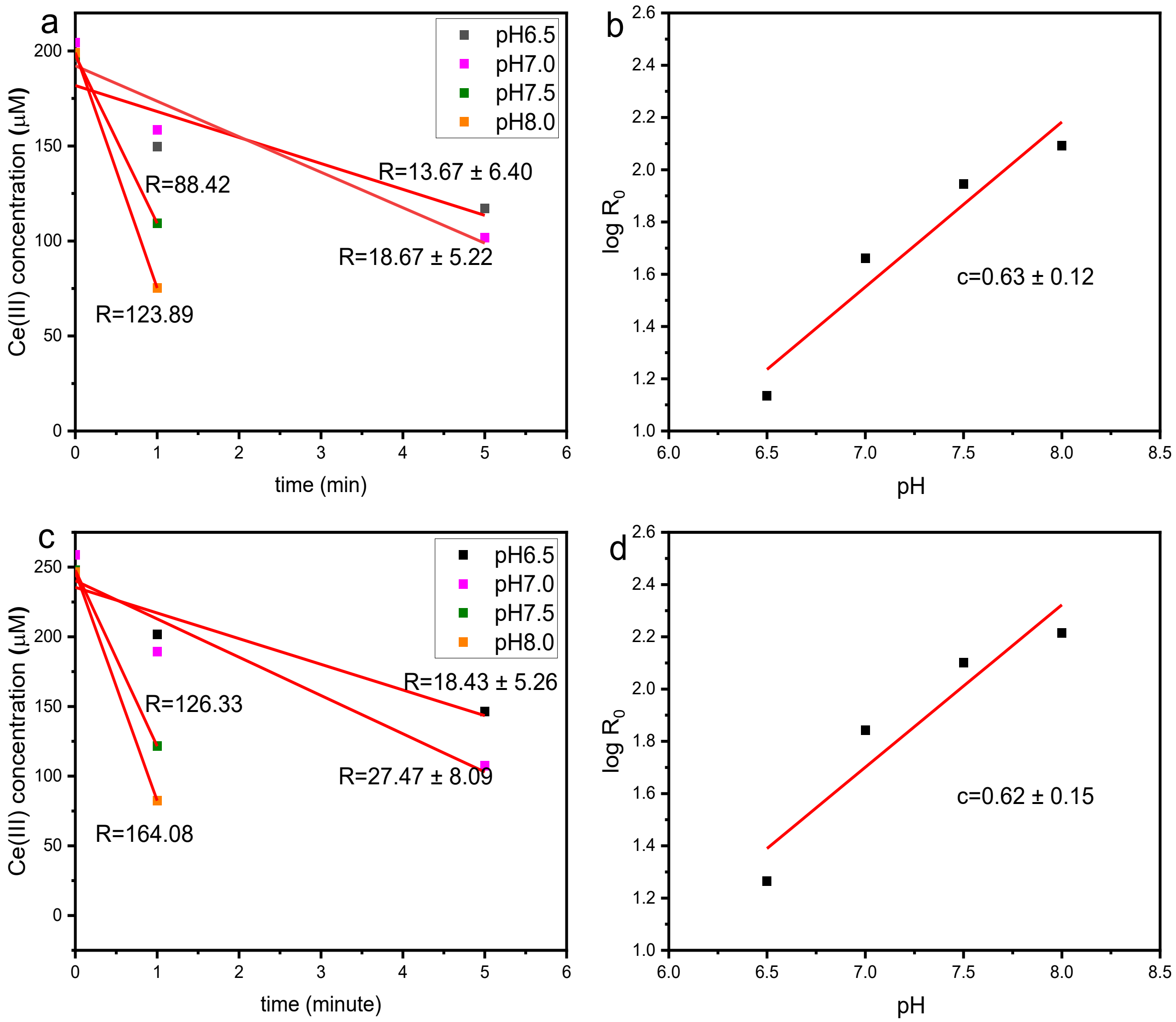

Figure S7. Results of replicate experiment to examine the reaction order with respect to pH at fixed δ-MnO_2 loads (0.1 g/L) and initial Ce(III) concentrations (200 μM for (a) and (b) and 250 μM for (c) and (d)).

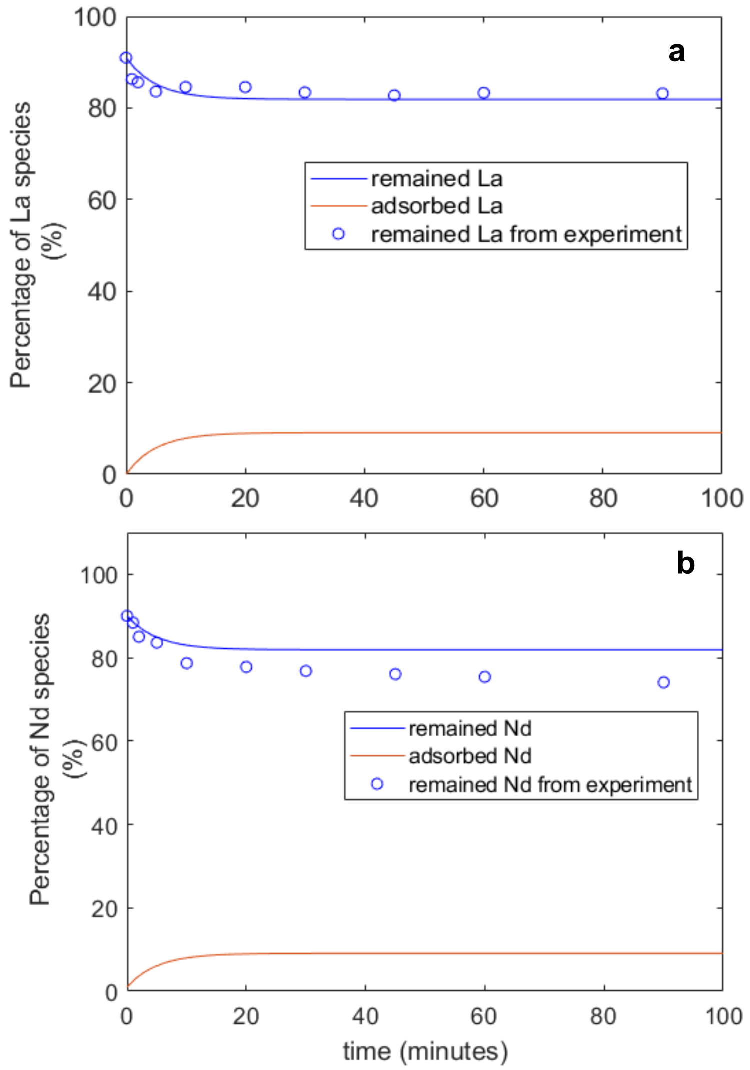

Figure S8. Ce(III) adsorption to δ-MnO_2 deduced from La(III) and Nd (III) adsorption to δ-MnO_2 and modeled constants. Fitting coefficient provided by corrcoef function is 0.8988 for La, and for 0.9693 for Nd.

References:

(1) Jung, H., Taillefert, M., Sun, J., Wang, Q., Borkiewicz, O. J., Liu, P., Yang, L., Chen, S., Chen, H., Tang, Y. Redox Cycling Driven Transformation of Layered Manganese Oxides to Tunnel Structures. *Journal of the American Chemical Society* **2020**, *142* (5), 2506-2513.

(2) Wan, B., Yang, P., Jung, H., Zhu, M., Diaz, J. M., Tang, Y. Iron Oxides Catalyze the Hydrolysis of Polyphosphate and Precipitation of Calcium Phosphate Minerals. *Geochimica et Cosmochimica Acta* **2021**, *305*, 49-65.
